# Editorial: Sex Differences in Molecular Mechanisms of Cardiovascular Aging

**DOI:** 10.3389/fragi.2022.854714

**Published:** 2022-03-23

**Authors:** S. Novella, K. L. Moreau, A. P. Dantas

**Affiliations:** ^1^ Department of Physiology, INCLIVA Biomedical Research Institute, University of Valencia, Valencia, Spain; ^2^ University of Colorado, Anschutz Medical Campus, Aurora, CO, United States; ^3^ Institut d’Investigacions Biomèdiques August Pi i Sunyer (IDIBAPS), Barcelona, Spain

**Keywords:** cardiovascular aging, sex difference, menopause, andropause, epigenetics

Modern society is facing a social, economic and public health challenge with the increase of an aging population. Aging is associated with an increase in the incidence of cardiovascular diseases (CVD), the leading cause of morbidity and mortality in both men and women, through adverse effects on the vasculature, as well as direct effects on the heart (Lakatta and Levy, 2003). There is a sex disparity in the development of CVD, with women having a lower prevalence of CVD than men up until midlife, where after prevalence rates become similar. Thus, sex-specific differences in cardiovascular aging may play an important role in the development of CVD. Although increasing efforts have been made in this regard, understanding how biological sex influences cardiovascular aging is imperative for meeting the challenges posed by a growing aging population.

This research topic presents a Research Topic of original research and reviews that address different aspects of the influence of sex and sex hormones on the mechanisms underlying cardiovascular aging. An in-depth review by Dela Justina et al. emphasizes the importance of recognizing sex as a biological variable by discussing the influence of biological sex on cardiac and vascular function and structure, and molecular and cellular mechanisms related to oxidative stress, and inflammation. The authors encouraged researchers to include both sexes in all study designs and approaches and stressed the need to develop experimental animal models that allow scientists to differentiate the effects attributed to aging and those generated by sex or sex hormones in cardiovascular pathophysiology. In support of this idea, Barros et al. discuss the most common aging and senescence-accelerated animal models with a focus on sex differences in aging-associated vascular alterations (i.e., endothelial dysfunction, remodeling, oxidative stress and inflammation).

Hormonal changes throughout aging affect the immune system that plays a fundamental role in the pathogenesis of CVD. In this context, Echem and Akamine describe new findings regarding the link between sex-associated differences in the regulation of the expression and signaling of a crucial member of the innate immune system, toll-like receptors (TLRs).

Endothelial function declines with aging, and the endothelin (ET-1) system is among the endothelial factors that are differentially modulated by sex and plays a key role in the vascular aging process. ET-1 exerts direct effects through receptors within the vasculature and interacts with a number of other cardiovascular mediators, such as nitric oxide (NO), prostacyclin (PGI2), and thromboxane. In the interesting review by Kuczmarski et al., translational evidence is presented addressing cell models, experimental and human studies. The sex-specific differences in the expression of ET-1 receptor subtypes and function suggest that the ET-1 system is an important contributor in the development and progression of vascular aging and associated diseases. Antagonists of ET-1 receptor A are being used in clinics for pulmonary arterial hypertension (PAH), which has a higher prevalence in women than in men. In this regard, the review presented by Rodriguez-Arias and García-Álvarez remarks the impact of sex in both animal models and patients suffering from pulmonary hypertension. The authors discuss the estrogen paradox in PAH that could partially explain the sex differences in prevalence, prognosis, and response to treatment independent of other factors such as comorbidities or differences in clinical management of PAH.

Clinical evidence establishes a vascular protection in aging women conferred by sex hormones, particularly estradiol through direct actions on the vascular endothelium. This effect is primarily mediated by estrogen receptor α (ERα) through genomic regulation of endothelium-derived vasoactive factors, as well as by non-genomic activation of rapid membrane-initiated steroid signaling. The thorough review written by Davezac et al. presents new insight into the mechanisms of action of estradiol acting on ERα in clinical and experimental studies reporting the protective effects of sex hormones on the arterial wall. In this review the authors discuss how abnormalities in the expression and/or function of ERs in the vasculature could contribute to the failure of estrogen signaling and vascular protection with aging, and therefore, selective modulation of ERα may optimize menopausal hormone therapy.

Controversy generated by menopausal hormone therapy in women has led to the establishment of the “timing hypothesis” (Clarkson et al., 2013), which states that the beneficial effects of estrogen therapy are diminished (or lost) in postmenopausal women that are greater than 10 years since the onset of menopause. Furthermore, environmental factors may contribute to a more or less favorable cardiovascular outcome of hormone therapy in postmenopausal women. In an original research paper, Hoier et al. describe how high intensity aerobic exercise training improves parameters of cardiovascular health in postmenopausal women that are >10 years since menopause. Although there was no improvement in popliteal artery endothelial function after the exercise intervention, cardiovascular risk profile was improved as indicated by a decrease in adiposity, blood pressure and increases in high-density lipoprotein (HDL) and maximal aerobic capacity. Moreover, the expression of skeletal muscle ERα and endothelial nitric oxide synthase (eNOS) expression was higher after the training intervention. These findings suggest that high intensity aerobic exercise training can be recommended for beneficial cardiovascular adaptations in healthy postmenopausal women that are greater than 10 years since menopause, a group of women who may not observe vascular protection with estrogen.

At the level of intracellular signaling, the original research conducted by Wang et al. shows sex- and age-dependent differences of phosphodiesterase PDE1-5 activities in the microvasculature of rat skeletal muscle. PDE controls cellular levels of cAMP and cGMP which in turn regulate multiple vascular functions. This basic research is central to designing new therapeutic strategies targeting PDE and moving towards personalized treatment in aging men and women.

Overall, the articles covered in this timely Research Topic provide an update, and also highlight gaps in knowledge on the effects of sex and sex hormones on the molecular and cellular mechanisms underlying cardiovascular aging that provides the foundation for future investigations.
